# Impact of the COVID-19 Pandemic on the Health Status and Behaviors of Adults in Korea: National Cross-sectional Web-Based Self-report Survey

**DOI:** 10.2196/31635

**Published:** 2021-11-26

**Authors:** EunKyo Kang, Hyejin Lee, Jee Hoon Sohn, Jieun Yun, Jin Yong Lee, Yun-Chul Hong

**Affiliations:** 1 National Cancer Control Institute National Cancer Center Goyang Republic of Korea; 2 Department of Family Medicine National Cancer Center Goyang Republic of Korea; 3 Department of Family Medicine Seoul National University Bundang Hospital Seongnam Republic of Korea; 4 Public Healthcare Center Seoul National University Hospital Seoul Republic of Korea; 5 Department of Psychiatrics Seoul National University Hospital Seoul Republic of Korea; 6 Department of Pharmaceutical Engineering Cheongju University Cheongju Republic of Korea; 7 Department of Health Policy and Management Seoul National University College of Medicine Seoul Republic of Korea; 8 Health Insurance Review and Assessment Service Research Institute Health Insurance Review and Assessment Service Wonju Republic of Korea; 9 Department of Preventive Medicine Seoul National University College of Medicine Seoul Republic of Korea; 10 Institute of Environmental Medicine Seoul National University Medical Research Center Seoul Republic of Korea

**Keywords:** COVID-19, health status, health behavior, self-reported online survey, pandemic, epidemiology, public health, sociodemographic factors, health interventions, lockdown

## Abstract

**Background:**

The COVID-19 pandemic has radically shifted living practices, thereby influencing changes in the health status and behaviors of every person.

**Objective:**

The aim of this study was to investigate the impact of COVID-19 on the self-reported health status and health behaviors along with any associated factors in adults in the Republic of Korea wherein no stringent lockdown measures were implemented during the pandemic.

**Methods:**

We conducted a web-based self-reported survey from November 2020 to December 2020. The study participants (N=2097) were identified through quota sampling by age, sex, and geographical regions among residents aged 19 years or older in Korea. The survey collected information on basic demographics, changes in self-reported health status, and health behaviors during the COVID-19 pandemic. Self-reported health status and health behaviors were categorized into 3 groups: unchanged, improved, or worsened. A chi-square test and logistic regression analyses were conducted.

**Results:**

With regard to changes in the self-reported health status, the majority (1478/2097, 70.5%) of the participants reported that their health was unchanged, while 20% (420/2097) of the participants reported having worser health after the COVID-19 outbreak. With regard to changes in health behaviors, the proportion of participants who increased tobacco consumption was similar to that of those who decreased tobacco consumption (110/545, 20.2% vs 106/545, 19.5%, respectively), while the proportion of those who decreased their drinking frequency was more than twice as many as those who increased their drinking frequency (578/1603, 36.1% vs 270/1603, 16.8%, respectively). Further, those who decreased their exercising frequency were more than those who increased their exercising frequency (333/823, 15.9% vs 211/823, 10%, respectively). The factor that had the greatest influence on lifestyle was age. In the subgroup analysis, the group aged 20-29 years had the highest number of individuals with both a worsened (100/377, 26.5%) and an improved (218/377, 15.7%) health status. Further, individuals aged 20-29 years had greater odds of increased smoking (6.44, 95% CI 2.15-19.32), increased alcohol use (4.64, 95% CI 2.60-8.28), and decreased moderate or higher intensity aerobic exercise (3.39, 95% CI 1.82-6.33) compared to individuals aged 60 years and older. Younger adults showed deteriorated health behaviors, while older adults showed improved health behaviors.

**Conclusions:**

The health status and the behavior of the majority of the Koreans were not found to be heavily affected by the COVID-19 outbreak. However, in some cases, changes in health status or health behavior were identified. This study highlighted that some groups were overwhelmingly affected by COVID-19 compared to others. Certain groups reported experiencing both worsening and improving health, while other groups reported unchanged health status. Age was the most influential factor for behavior change; in particular, the younger generation’s negative health behaviors need more attention in terms of public health. As COVID-19 prolongs, public health interventions for vulnerable groups may be needed.

## Introduction

The COVID-19 pandemic has radically shifted living practices around the world. Governments have implemented social distancing measures, urged employees to work from home, and banned mass gatherings to prevent the spread of COVID-19 [1,2]. Individuals have been isolated owing to self-quarantine measures in the case of suspected or confirmed COVID-19 cases [1,2]. Social isolation and restrictions on daily activities influence health behaviors such as smoking, alcohol consumption, and physical activity [3-19]. This contributes to mental health illnesses [18] and physical health problems, including chronic diseases, which may worsen the overall health status [19].

Several studies have been conducted regarding the impact of the COVID-19 pandemic on health status and behaviors. Previous studies have shown that most of the health statuses, including self-reported physical health and mental health, tended to worsen [11] and health behaviors also deteriorated due to COVID-19. Several studies have shown that there has been an increase in the amount of smoking [3-5], an increase in relapse to smoking [6], and an increase in alcohol consumption [3,7,9-12,20] during the COVID-19 pandemic. Binge-eating behaviors and reduced level of exercise have also been reported [13], suggesting that the COVID-19 pandemic has led to poor health behaviors. However, positive behavioral changes have also been reported in some studies, such as people who were less active before the COVID-19 pandemic performing more exercises [14], or trying to quit smoking [15,16], or smoking less during the lockdown periods [17].

Previous studies have reported that social isolation during COVID-19 generally had an unfavorable impact on health status and behaviors; however, it also motivated individuals to take self-guided actions to improve their health. Although changes in health status and health behavior are complex and multifaceted, these previous studies have focused on specific populations [4,18], extreme circumstances such as lockdown or disasters [3,5,12,14,15], and certain health conditions and health behaviors [6,10,11,13,17]. Therefore, in the ongoing COVID-19 situation, it was difficult to understand the overall changes in health status and behavior caused by the restriction of physical activity and mental stress. However, it is important to identify the factors related to changes in health status and behaviors in terms of identifying high-risk groups requiring intervention. Several studies have reported that demographic factors, socioeconomic level, residential area, and disease status are related to lifestyle changes due to COVID-19 [21-23]. The objective of this study was to investigate whether the COVID-19 pandemic has induced changes in self-reported health status and health behaviors in 2020 in a country without stringent lockdown measures. We also identified the factors associated with changes in self-reported health status and health behaviors such as smoking, alcohol consumption, and exercise.

## Methods

### Participants and Recruitment

This cross-sectional survey was initiated on November 9, 2020. At the time of the survey, the total population of Korea according to the 2020 census by Statistics Korea was 51,780,000 [24], and we tried to calculate a representative sample size on behalf of the Korean population. Assuming 95% CI, 2.2% margin of error, and standard deviation of 0.5%, the estimated sample size was 1984, and we decided to gather more than 2000 participants. The total study participant recruitment period took 4 weeks from November 9 to December 4, 2020. This study was performed with technical support from Gallup Korea, a global social research company, and conducted in all regions of Korea. The study participants were identified through quota sampling by age, sex, and geographical regions among residents aged 19 years and older in the Republic of Korea. Korea is distributed geographically into 16 administrative distributes. Based on the 2015 National Statistical Office census data, this study was designed to sample 2000 people according to the population structure and a systematic random sampling method was implemented. In the web-based survey, the appropriate number of samples was allocated according to the population distribution for each of the 16 administrative district units, and the number of participants by age and gender was assigned to each unit. The estimated sample error of our study was 2.5% at 95% CI.

The survey was conducted as follows: the study participants were informed of the purpose of the study, they consented to participate, and they were directed to an encrypted website to complete the survey. The study was conducted anonymously, but to prevent duplicate questionnaires, the study participants used a mobile phone–based verification system. When the respondents enter their cell phone number before responding, the passcode is transmitted, and the passcode is used to identify the owner of the cell phone based on the information of the telecommunication company, thereby avoiding duplicate questionnaires. The questionnaire in this study consisted of multiple-choice questions, and there were no missing values in the case of the respondents who completed the survey because it was impossible to move to the next page if a response was omitted. Further, the questionnaire on 1 page consisted of about 10 questions on average, and if the study participant answered the same or similar answers to the questions on 1 page, a pop-up was set up to confirm whether the answers were certain before moving on to the next page. As a result, 1280 people dropped out of the study, including those who declined during the survey or refused to respond to the survey before they initiated the survey. Of the 3377 participants who consented to partake, 2097 completed the study and the dropout rate in the study was 37.9% (2097/3377). Those who completed the survey received an electronic device worth US $5.

The inclusion criteria included being ≥19 years old and physically healthy and mentally stable enough to read, understand, and answer the questionnaire. Further, to sample a representative group representing Koreans, we excluded those who were not residing in Korea from the study. Those who did not speak Korean were excluded from the study because they could have limitations in understanding the consent form or in reading and answering questions. The study protocol was approved by the Institutional Review Board of Seoul National University Hospital (IRB E-2011-102-1173). This study follows the guidelines of the STROBE (STrengthening the Reporting of OBservational studies in Epidemiology) checklist.

### Survey Measures

The web-based survey collected information on basic demographics, changes in self-reported health status, and health behaviors during the COVID-19 pandemic. The demographic variables included sex, age, region, household income, education level, supplementary private health insurance, marital status, occupation, and presence of chronic diseases. Participants were categorized into 5 age groups (20-29 years, 30-39 years, 40-49 years, 50-59 years, and >60 years), and 3 groups as per the area of residence (Seoul metropolitan area, Daegu-Gyeongbuk province, and others) according to the incidence of COVID-19 epidemic in the area. Household income was classified into 4 groups (US $2000, US $2000-$3999, US $4000-$5999, ≥US $6000), and education level was categorized into 3 groups (high school graduate and undergraduate, college/university graduate or associate degree, and master’s degree or above). Lastly, marital status was categorized into 3 groups (single, married, and divorced or widowed), while occupation was categorized into 4 groups (office worker, manual worker, self-employed, and housewife/student/unemployed).

In accordance with the previous studies on self-reported health status [25,26], the study participants were asked to report their health status 1 year before (prior to the COVID-19 outbreak) and their current health status (after the COVID-19 outbreak) to identify the changes associated with the COVID-19 pandemic. Questions about self-reported health status were obtained by using the original questionnaire of short form-36 items, and reference time was added as a footnote to reduce the recall bias of respondents. Self-reported health status was measured on a 5-point Likert scale from 1 (poor) to 5 (excellent) and then further categorized into 3 groups (unchanged, improved, or worsened). “Unchanged” was defined as a case in which the responses before and after were identical, “improved” as a case in which the health status improved after compared to before, and “worsened” as a case where the health status deteriorated after compared to before. Questions on smoking, alcohol consumption, and moderate or higher intensity aerobic exercise were partially derived from the Centers for Disease Control and Prevention’s National Health Interview Survey. We had to generate questions about the changes before and after the COVID-19 outbreak for our study, and for the quantitative comparison of changes, we had to separately analyze the amount of smoking, drinking, and exercise before and after the COVID-19 outbreak. Detailed questions are provided separately in Multimedia Appendix 1. The changes in smoking, drinking, and exercise were classified into 3 groups (increased, decreased, or unchanged) through questions on the amount of smoking, drinking, and exercise 1 year before (before the onset of COVID-19) and now.

### Statistical Analysis

A chi-square test was performed to compare the categorical variables such as changes in health status, smoking, alcohol consumption, and exercise at moderate intensity or higher before and after the COVID-19 outbreak. Logistic regression analyses were conducted to identify the factors associated with improving or worsening health behaviors. Demographic factors included in these regression analyses were sex, age, region, household income, education levels, supplementary private health insurance, marital status, occupation, and presence of chronic diseases. The results of the logistic regression analyses were presented with 95% CIs and adjusted odds ratio (aOR). Statistical significance was defined as a two-tailed *P* value <.05. All statistical analyses were performed using Stata version 23 (StataCorp LLC).

## Results

### Baseline Characteristics of the Participants

Table 1 shows the baseline characteristics of the participants. Of the 2097 participants, 1058 (50.5%) were men and 1039 (49.5%) were women. The study participants were evenly distributed across 5 age groups. Among them, 401 (19.1%) lived in the Seoul metropolitan area, 196 (9.4%) lived in the Daegu-Gyeongbuk province, and 1500 (71.5%) lived in other areas. Of the 2097 participants, 192 (9.2%) had an household income <US $2000, 1498 (71.4%) were university graduates, 1718 (81.9%) held supplementary private health insurance, 755 (36%) were singles, 1251 (59.7%) were married, 1110 (52.9%) were office workers, followed by 582 (27.8%) housewives, students, or unemployed, and 1081 (51.6%) participants had more than one preexisting chronic condition. Among the 2097 participants, 545 (26%) were smokers and 1603 (76.4%) regularly consumed alcohol (Table 1).

**Table 1:** Baseline characteristics of the participants (N=2097).

Variable		Values, n (%)
**Sex**		
	Men	1058 (50.5)
	Women	1039 (49.5)
**Age (years)**		
	20-29	377 (18)
	30-39	411 (19.6)
	40-49	485 (23.1)
	50-59	479 (22.8)
	≥60	345 (16.5)
**Region**		
	Seoul metropolitan area	401 (19.1)
	Daegu-Gyeongbuk province	196 (9.4)
	Others	1500 (71.5)
**Household income (USD)**		
	≤$2000	192 (9.2)
	$2000-$3999	684 (32.8)
	$4000-$5999	610 (29.2)
	≥$6000	600 (28.8)
**Educational status**		
	High school graduate and under	359 (17.2)
	College/university graduation or associate degree	1498 (71.4)
	Master's degree or above	240 (11.4)
**Supplementary** **private insurance**		
	Yes	1718 (81.9)
	No	379 (18.1)
**Marital status**		
	Single	755 (36)
	Married	1251 (59.7)
	Widowed/divorced	91 (4.3)
**Job**		
	Office worker	1110 (52.9)
	Manual worker	212 (10.1)
	Own business	193 (9.2)
	Housewife/student/unemployed	582 (27.8)
**Chronic illness**		
	Yes	1081 (51.6)
	No	1016 (48.5)
**Change of health status**		
	Getting worse	420 (20)
	Unchanged	1478 (70.5)
	Getting better	199 (9.5)
**Smoking**		
	Yes	545 (26)
	No	1552 (74)
**Drinking**		
	Yes	1603 (76.4)
	No	494 (23.6)

### Changes in Health Status and Behaviors During the COVID-19 Pandemic

**Figure 1:**
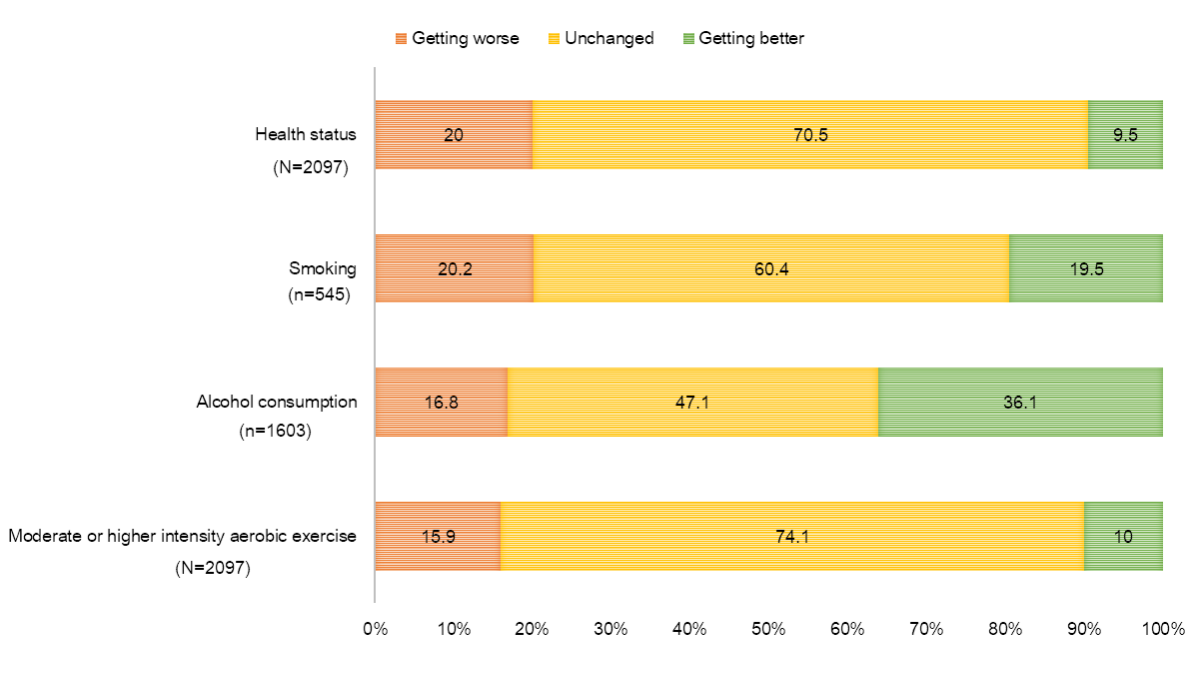
Changes in the health status and behaviors of the adults in Korea during the COVID-19 pandemic.

Regarding changes in health status before and after COVID-19, 1478 (70.5%) of the 2097 participants reported their health was unchanged, and 420 (20%) participants reported that their health status was worser than before. Among the 2097 participants, 199 (9.5%) responded that their health status improved. When asked about health behavior, among 545 smokers, 110 (20.2%) reported that their smoking frequency had increased while 106 (19.5%) reported that their smoking frequency had decreased, and these proportions were almost similar. With regard to drinking, 578 (36.1%) participants reported a decrease in drinking frequency while 270 (16.8%) reporting an increase—more than double the number of people decreased their drinking frequency. Decreased moderate or high-intensity aerobic exercise was reported by 333 (15.9%) participants whereas 211 (10%) reported an increased frequency in exercising after the onset of the pandemic (Figure 1).

### Changes in Self-reported Health Status During the COVID-19 Pandemic in Subgroup Analyses

**Figure 2:**
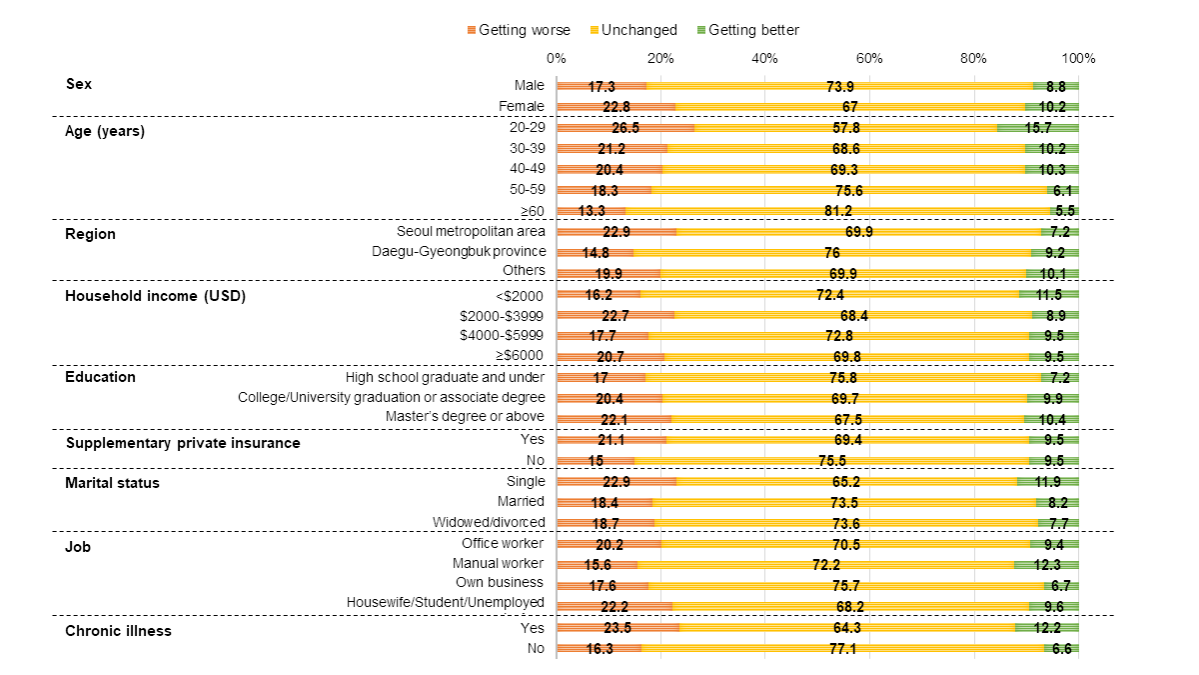
Changes in the health status of the adults in Korea by subgroup during the COVID-19 pandemic. The change in health status was surveyed by 2097 participants.

Women reported having both worser health (237/1039, 22.8%) and better health (106/1039, 10.2%) than men (183/1058, 17.3% and 93/1058, 8.8%, respectively; *P*<.001). Regarding age groups, the 20-29 years age group had the highest number of individuals with both a worsened (100/377, 26.5%) and an improved (59/377, 15.7%) health status. However, participants aged 60 years and older reported having the least of either worse (46/345, 13.3%) or improved (19/345, 5.5%) health status. Among the participants with supplementary private health insurance, 21.1% (363/1718) reported that their health had become worse (*P*=.03). We found that 22.9% (173/755) of the singles were more likely to gain a worser health status than those who were married (230/1251, 18.4%) and divorced or widowed (17/91, 18.7%). However, 11.9% (90/755) of the singles were more likely to have an improved health status than married (102/1251, 8.2%), divorced, or widowed (7/91, 7.7%) participants as well. Participants with preexisting chronic diseases (254/1081, 23.5%) reported having a worser health status more than those without (166/1016, 16.3%) (*P*<.001) (Figure 2).

### Factors Associated With Worsening Health Behaviors

Age was the main risk factor for increased smoking; the aOR of increased smoking was higher among participants aged 20-29 years (aOR 6.44, 95% CI 2.15-19.32) than that among those who were aged 60 years and older. Further, age was the greatest risk factor for increased alcohol use; the aOR of increased drinking was the highest among participants aged 30-39 years (aOR 4.64, 95% CI 2.60-8.28). Further, individuals aged 20-29 years (aOR 3.39, 95% CI 1.82-6.33) had greater odds of decreased moderate or higher intensity aerobic exercise than those aged 60 years and older. Participants with preexisting chronic diseases were more associated with worsening of all health behaviors than those without, revealing increased smoking (aOR 2.07, 95% CI 1.28-3.36), increased alcohol consumption (aOR 1.44, 95% CI 1.09-1.90), and decreased moderate or higher intensity exercise (aOR 1.51, 95% CI 1.28-1.79). The area of residence, lower monthly household income, higher education level, and marriage status were also associated with worsening health behaviors (Table 2).

**Table 2:** Factors associated with worsening health behaviors.^a^

Variable		Increased smoking^b^	Increased alcohol consumption^c^	Decreased moderate or higher intensity aerobic exercise^d^
**Sex**				
	Men	1.00 (Reference)	1.00 (Reference)	1.00 (Reference)
	Women	1.16 (0.66-2.02)	1.00 (0.75-1.32)	1.21 (0.90-1.65)
**Age (years)**				
	20-29	*6.44 (2.15-19.32)*	*3.23 (1.64-6.40)*	*3.39 (1.82-6.33)*
	30-39	*3.74 (1.52-9.25)*	*4.64 (2.60-8.28)*	*2.45 (1.41-4.27)*
	40-49	1.94 (0.82-4.63)	*3.98 (2.31-6.85)*	*2.02 (1.24-3.29)*
	50-59	1.69 (0.71-4.04)	*2.10 (1.21-3.66)*	1.53 (0.97-2.43)
	≥60	1.00 (Reference)	1.00 (Reference)	1.00 (Reference)
**Region**				
	Seoul metropolitan area	1.22 (0.72-2.05)	*1.74 (1.26-2.39)*	1.04 (0.73-1.48)
	Daegu-Gyeongbuk province	0.73 (0.33-1.61)	0.69 (0.40-1.18)	0.63 (0.35-1.11)
	Others	1.00 (Reference)	1.00 (Reference)	1.00 (Reference)
**Household income (USD)**				
	≤$2000	1.00 (Reference)	1.00 (Reference)	1.00 (Reference)
	$2000-$3999	0.70 (0.24-2.05)	1.01 (0.56-1.84)	1.23 (0.68-2.24)
	$4000-$5999	0.40 (0.13-1.22)	0.73 (0.39-1.35)	1.28 (0.69-2.36)
	≥$6000	*0.30 (0.10-0.94)*	0.90 (0.48-1.66)	1.20 (0.65-2.24)
**Educational status**				
	High school graduate and undergraduate	1.00 (Reference)	1.00 (Reference)	1.00 (Reference)
	College/university graduation or associate degree	0.90 (0.48-1.68)	0.86 (0.57-1.29)	*1.70 (1.09-2.64)*
	Master's degree or above	1.82 (0.77-4.26)	0.82 (0.47-1.44)	*2.23 (1.40-3.55)*
**Supplementary** **private insurance**				
	Yes	1.70 (0.83-3.51)	1.14 (0.76-1.73)	1.28 (0.85-1.94)
	No	1.00 (Reference)	1.00 (Reference)	1.00 (Reference)
**Marital status**				
	Single	1.00 (Reference)	1.00 (Reference)	1.00 (Reference)
	Married	1.62 (0.86-3.02)	*1.61 (1.10-2.34)*	1.34 (0.87-2.06)
	Widowed/divorced	*2.95 (1.01-8.66)*	1.94 (0.90-4.19)	1.42 (0.64-3.16)
**Job**				
	Office worker	1.00 (Reference)	1.00 (Reference)	1.00 (Reference)
	Manual worker	1.27 (0.65-2.50)	1.20 (0.74-1.94)	0.87 (0.52-1.48)
	Own business	1.29 (0.61-2.74)	*1.60 (1.09-2.20)*	1.52 (0.88-2.62)
	Housewife/student/ unemployed	0.59 (0.24-1.44)	0.86 (0.58-1.27)	1.29 (0.89-1.88)
**Chronic illness**				
	Yes	*2.07 (1.28-3.36)*	*1.44 (1.09-1.90)*	*1.51 (1.28-1.79)*
	No	1.00 (Reference)	1.00 (Reference)	1.00 (Reference)

^a^Values in italics indicate statistically significant values.

^b^Change in smoking behavior was surveyed by 545 current smokers.

^c^Change in alcohol consumption was surveyed by 1603 participants who answered that they regularly consumed alcohol.

^d^Change in exercise behavior was surveyed by 2097 participants.

### Factors Associated With Improving Health Behaviors

Regarding age, the aOR of decreased smoking for improving health behaviors was higher among participants aged 60 years and older (aOR 3.38, 95% CI 1.06-10.78) than that among those who were aged 20-29 years, whereas the aOR of increased moderate or higher intensity aerobic exercise for improving health behaviors was the highest among the latter. Higher monthly household income and education level, that is, those who had attained at most a high school diploma or less, were also associated with improving health behaviors (Table 3).

**Table 3:** Factors associated with improving health behaviors.^a^

Variable		Decreased smoking^b^	Decreased alcohol consumption^c^	Increased moderate or higher intensity aerobic exercise^d^
**Sex**				
	Men	1.00 (Reference)	1.00 (Reference)	1.00 (Reference)
	Women	1.35 (0.79-2.3)	1.14 (0.91-1.42)	1.19 (0.88-1.60)
**Age (years)**				
	20-29	1.00 (Reference)	1.00 (Reference)	1.00 (Reference)
	30-39	1.6 (0.66-3.88)	0.71 (0.49-1.02)	*0.55 (0.33-0.89)*
	40-49	1.71 (0.69-4.25)	0.7 (0.48-1.04)	*0.56 (0.33-0.95)*
	50-59	1.86 (0.72-4.82)	0.93 (0.61-1.41)	*0.50 (0.28-0.87)*
	≥60	*3.38 (1.06-10.78)*	0.99 (0.62-1.58)	0.54 (0.29-1.01)
**Region**				
	Seoul metropolitan area	0.97 (0.57-1.65)	0.81 (0.61-1.06)	0.69 (0.46-1.04)
	Daegu-Gyeongbuk province	0.94 (0.44-2.01)	0.83 (0.58-1.21)	1.14 (0.71-1.82)
	Others	1.00 (Reference)	1.00 (Reference)	1.00 (Reference)
**Household income (USD)**				
	≤$2000	1.00 (Reference)	1.00 (Reference)	1.00 (Reference)
	$2000-$3999	1.12 (0.35-3.53)	1.54 (0.97-2.45)	1.44 (0.97-2.13)
	$4000-$5999	1.05 (0.32-3.43)	*1.77 (1.10-2.84)*	1.45 (0.98-2.15)
	≥$6000	1.41 (0.43-4.62)	*1.91 (1.19-3.09)*	*2.12 (1.25-3.60)*
**Educational status**				
	High school graduate and undergraduate	*3.22 (1.18-8.79)*	0.81 (0.53-1.24)	0.94 (0.51-1.71)
	College/university graduation or associate degree	2.45 (0.99-6.04)	0.68 (0.49-0.94)	1.02 (0.63-1.63)
	Master's degree or above	1.00 (Reference)	1.00 (Reference)	1.00 (Reference)
**Supplementary** **private insurance**				
	Yes	1.00 (Reference)	1.00 (Reference)	1.00 (Reference)
	No	0.67 (0.33-1.34)	1.15 (0.86-1.55)	0.82 (0.54-1.24)
**Marital status**				
	Single	1.00 (Reference)	1.00 (Reference)	1.00 (Reference)
	Married	0.78 (0.42-1.45)	*0.68 (0.50-0.92)*	1.12 (0.73-1.73)
	Widowed/divorced	0.21 (0.04-1.05)	0.53 (0.28-1.02)	0.72 (0.28-1.82)
**Job**				
	Office worker	1.00 (Reference)	1.00 (Reference)	1.00 (Reference)
	Manual worker	1.65 (0.88-3.12)	1.32 (0.91-1.91)	0.73 (0.40-1.32)
	Own business	0.72 (0.33-1.57)	0.77 (0.51-1.15)	1.43 (0.87-2.37)
	Housewife/student/unemployed	1.53 (0.70-3.30)	0.95 (0.72-1.27)	0.85 (0.58-1.24)
**Chronic illness**				
	Yes	1.15 (0.73-1.81)	1.10 (0.89-1.37)	0.98 (0.73-1.32)
	No	1.00 (Reference)	1.00 (Reference)	1.00 (Reference)

^a^Values in italics indicate statistically significant values.

^b^Change in smoking behavior was surveyed by 545 current smokers.

^c^Change in alcohol consumption was surveyed by 1603 participants who answered that they regularly consumed alcohol.

^d^Change in exercise behavior was surveyed by 2097 participants.

## Discussion

This study investigated whether the COVID-19 pandemic induced changes in self-reported health status and behaviors in adults in Korea, wherein stringent lockdown measures were not implemented. Most participants in this study reported an unchanged health status around 1 year before and after the COVID-19 epidemic. More participants reported that they became worse than those who reported they became better, which is consistent with the results of previous studies [11]. Both self-reported worsening and improving of health status were common in younger age groups. In addition, young age was a major risk factor for deterioration of health behaviors. From this, it is estimated that specific populations were experiencing varied impacts whereas other groups were not affected at all during the pandemic.

Contrary to previous studies that found an increased prevalence of smoking during the COVID-19 pandemic [3-5], this study found that previously identified increases in smoking among smokers were, in most cases, unchanged from prepandemic levels. Several studies showed increased alcohol consumption during the initial phase of the pandemic [10-12,27]; however, after social distancing measures were adopted, studies noted a reduction in alcohol consumption [8,28]. Similarly, our results reported that more participants had decreased alcohol intake than those who had increased alcohol intake during the pandemic.

Tailoring public health responses to targeted groups may be important for mitigating health behavioral problems during the COVID-19 pandemic. Previous studies showed that smoking was associated with an increased risk of COVID-19 progression [29,30]. Thus, it is important to identify the population groups who are more likely to engage in smoking as part of the COVID-19 risk management at the population level. This study revealed that reduction in smoking was associated with older age whereas an increase in smoking was associated with younger age, which aligned with previous findings that older smokers were more likely to quit than younger smokers during the pandemic [22]. Furthermore, being divorced or separated was also associated with an increase in smoking [22,31]. In contrast with what was previously found, financially stable individuals were less likely to smoke more [31], whereas highly educated individuals were reported to be less sensitive to reducing the frequency of their smoking, which aligned with that reported in earlier studies [32]. Lastly, preexisting chronic diseases were shown to be associated with increased smoking, as noted in previous studies [33,34].

Marital status had a significant association with alcohol consumption, showing an increased level for couples that were married. This was contrary to previous findings that single [35] and younger individuals [21,36] were more likely to consume more alcohol. Interestingly, individuals with a higher household income were more likely to report decreased alcohol consumption during the pandemic. This was in contrast to previous findings from the United Kingdom and the Pan American Health Organization that reported individuals with a higher household income had an increase in alcohol use [21,37]. Individuals living in the areas hit harder by COVID-19 were found to drink with higher frequency, which may be explained by the fear of COVID-19 infection [21]. Moreover, increased drinking during the pandemic was associated with psychological factors such as stress and noncompliance with social distancing measures as reported by a previous study [38]. Other factors, including self-employment and the presence of preexisting chronic conditions, were associated with an increase in alcohol use during the COVID-19 pandemic, aligning with an earlier study that demonstrated that job insecurity was more prevalent in those who were self-employed [23]. Furthermore, being married was also associated with a reduction in alcohol use. These results differ from those of previous studies that suggested single people drank more or that marital status was not associated with changes in alcohol consumption [9,35,36].

The COVID-19 pandemic might significantly affect body weight–related behaviors, including reduced levels of physical activity [39,40]. In particular, it was previously reported that decreased physical activity was remarkably widespread among obese populations and a higher body mass index was associated with an increased risk for COVID-19 hospitalizations and deaths [41-43]. This highlights the importance of physical activity for all groups during the COVID-19 pandemic. We found that amounts of exercise increased in the middle-aged populations but decreased in the younger age groups. Mandated work-from-home conditions may have contributed to the drop in physical activity and exercise among younger groups, while exercise promotions among middle-aged groups may have contributed to their consequential increase [44]. Individuals with college or university degrees or above were more likely to experience a reduction in exercise during the pandemic compared to those with a high school diploma or lower academic degree. This demonstrates that individuals with advanced degrees can be targeted for improvement in physical activities [45]. In contrast to the previous research that suggested individuals from higher-income households experienced a decrease in weight gain protective behaviors [46], we found that individuals with higher-income households actually experienced more of these behaviors during the pandemic. Lastly, having a preexisting chronic condition was significantly associated with reduced exercise. The chronic disease itself may cause a lack of physical activity; therefore, more attention should be paid to increasing regular exercise among patients with chronic diseases, especially during this pandemic.

The results on the health status and behaviors clearly outline the implications for public health both before and after the COVID-19 outbreak. Although the impact of the pandemic on health behaviors may vary widely by population group, the majority of the people did not notice significant changes. However, in some risk groups, health behaviors worsened, such as exercising less, drinking more alcohol, and smoking more cigarettes. Taken together, these findings demonstrate a need for public health interventions to manage health behaviors as the COVID-19 pandemic continues. Additionally, there is a need for follow-ups for those with chronic diseases to better understand their changes in health status and behaviors during the COVID-19 period.

Our study has several limitations. First, as participants were asked to compare their health status and behaviors 1 year ago to their current status and behaviors, recall bias may happen. In addition to this, intentional distortion of answers could not be ruled out. To reduce the recall bias as much as possible when scheming the items, the reference points were presented. Since most of the participants responded in November 2020, rather than simply suggesting “1 year ago,” the footnote below the question indicated “November 2019, before the outbreak of COVID-19 outbreak.” “The present” is also indicated as November 2020, after the COVID-19 outbreak. Second, since it is a self-report questionnaire, the results may vary depending on the person's perception. Third, since this study is a cross-sectional study, the causal relationship is unknown. Finally, since we only included a representative sample of the Korean population, our results may not be generalizable to other populations. Considering that Korea has never implemented lockdown measures, this study has successfully identified overall changes in health status and behavior and related factors during the pandemic under nonextreme circumstances. In addition, this study is meaningful in providing information on groups vulnerable to changes in health behavior due to COVID-19.

In conclusion, this study identified changes in health status and behavior and factors related to changes before and after the onset of COVID-19 in addition to confirming the characteristics of the group with worsened health status and behaviors. In particular, younger generation’s negative health behaviors need more attention in terms of public health. As COVID-19 prolongs, public health interventions for vulnerable groups may be needed.

## Appendix

Multimedia Appendix 1 Self-reported health status questionnaire.

## Abbreviations

aOR: adjusted odds ratio
STROBE: STrengthening the Reporting of OBservational studies in Epidemiology
