# Supplementary material for: Impact of the COVID-19 Pandemic on the Health Status and Behaviors of Adults in Korea: National Cross-sectional Web-Based Self-report Survey
Source: JMIR Public Health Surveill. 2021 Nov 26;7(11):e31635. doi: 10.2196/31635 (PMC8629347; doi:10.2196/31635)
Supplement: Multimedia Appendix 1 [file publichealth_v7i11e31635_app1.docx]

**Q1. Self-reported health status**

**Q1-1.** How would you rate your health in general now*?

*November 2020, after the COVID-19 outbreak

1) Excellent 2) Very Good 3) Good

4) Fair 5) Poor

**Q1-2.** Compared to now, how would you rate your health a year ago*?

*November 2019, before the COVID-19 outbreak

1) Excellent 2) Very Good 3) Good

4) Fair 5) Poor

**Q2. Health behaviors – smoking**

**Q2-1.** Do you currently smoke cigarettes?

1) Yes 2) No

→ If “No,” go **Q 2-1-1**, read question below. After answering the **Q 2-1-1**, go to **Q3** and continue the survey.

→ If “yes,” go **Q 2-2**, read each question below **Q 2-2**. For each question, enter the answer choice which best describes your response.

**Q2-1-1.** Did you previously smoke cigarettes?

1) Yes (Former smoker) 2) No (Never smoker)

**Q2-2.** Do you now smoke cigarettes every day or some days?

1) Every day 2) Some days

**Q2-3.** Compared to November 2019 (before the outbreak of COVID-19), is there any change in your smoking amount?

1) Increased 2) Decreased 3) No change 4) Don’t know

**Q2-4-1.** How many cigarettes did you smoke per day a year ago*?

*November 2019, before the COVID-19 outbreak

**( )** cigarettes / day

**Q2-4-2.** How many cigarettes did you smoke per day now*?

*November 2020, after the COVID-19 outbreak

**( )** cigarettes / day

**Q3.** **Health behaviors – alcohol consumption**

**Q3-1.** How often do you have a drink containing alcohol?

1) Never 2) Monthly or less 3) 2 to 4 times a month

4) 2 to 3 times a week 5) 4 or more times a week

→ If “Never,” go **Q4**, read question below.

**Q3-2.** How many drinks* containing alcohols do you have on a typical day when you are drinking?

*The standard for the number of drinking glasses for each alcoholic beverage converted by considering the alcohol content of each alcoholic beverage, such as soju, bottled beer, canned beer, draft beer, makgeolli, and wine, is presented in a table.

Types of alcoholic beverages mainly consumed: ( )

( ) drink(s)/day

**Q3-3.** Compared to November 2019 (before the outbreak of COVID-19), is there any change in your drinking amount or frequency?

1) Increased 2) Decreased 3) No change 4) Don’t know

**Q3-4-1.** How often did you have a drink containing alcohol a year ago*? If it is less than once a week, please answer ‘per month’, and if it is more than once a week, please answer ‘per week’.

*November 2019, before the COVID-19 outbreak

**( )** / month or **( )** / week

**Q3-4-2.** How many drinks containing alcohols did you have on a typical day when you were drinking a year ago*?

*November 2019, before the COVID-19 outbreak

Types of alcoholic beverages mainly consumed: ( )

( ) drink(s)/day

**Q3-4-3.** How often do you have a drink containing alcohol now*? If it is less than once a week, please answer ‘per month’, and if it is more than once a week, please answer ‘per week’.

*November 2020, after the COVID-19 outbreak

**( )** / month or **( )** / week

**Q3-4-4.** How many drinks containing alcohols do you have on a typical day when you are drinking now*?

*November 2020, after the COVID-19 outbreak

Types of alcoholic beverages mainly consumed: ( )

( ) drink(s)/day

**Q4.** **Health behaviors – moderate* or higher** intensity aerobic exercise**

*Moderate-intensity aerobic activities are activities that slightly increase in breathing or heart rate, such as brisk walking. Examples include: Brisk walking (at least 2.5 miles per hour), water aerobics, dancing (ballroom or social), gardening, tennis (doubles), biking slower than 10 miles per hour

**Q4-1-1.** In a typical week, on how many days do you do moderate-intensity activities now*?

*November 2020, after the COVID-19 outbreak

( ) day(s) / week

**Q4-1-2.** How much time do you spend doing moderate-intensity activities on a typical day now*?

*November 2020, after the COVID-19 outbreak

( ) minutes / day

**Q4-2-1.** In a typical week, on how many days did you do moderate-intensity activities a year ago*?

*November 2019, before the COVID-19 outbreak

( ) day(s) / week

**Q4-2-2.** How much time did you spend doing moderate-intensity activities on a typical day a year ago*?

*November 2019, before the COVID-19 outbreak

( ) minutes / day

**Higher-intensity aerobic activities are activities that large increases in breathing or heart rate, such as hiking uphill or running. Examples include: Hiking uphill or with a heavy backpack, running, swimming laps, aerobic dancing, heavy yardwork like continuous digging or hoeing, tennis (singles), cycling 10 miles per hour or faster, jumping rope

**Q4-3-1.** In a typical week, on how many days do you do higher-intensity activities now*?

*November 2020, after the COVID-19 outbreak

( ) day(s) / week

**Q4-3-2.** How much time do you spend doing higher-intensity activities on a typical day now*?

*November 2020, after the COVID-19 outbreak

( ) minutes / day

**Q4-4-1.** In a typical week, on how many days did you do higher-intensity activities a year ago*?

*November 2019, before the COVID-19 outbreak

( ) day(s) / week

**Q4-4-2.** How much time did you spend doing higher-intensity activities on a typical day a year ago*?

*November 2019, before the COVID-19 outbreak

( ) minutes / day
